# A Genome-Based Species Taxonomy of the *Lactobacillus* Genus Complex

**DOI:** 10.1128/mSystems.00264-19

**Published:** 2019-09-03

**Authors:** Stijn Wittouck, Sander Wuyts, Conor J. Meehan, Vera van Noort, Sarah Lebeer

**Affiliations:** a Research Group Environmental Ecology and Applied Microbiology, Department of Bioscience Engineering, University of Antwerp, Antwerp, Belgium; b Centre of Microbial and Plant Genetics, KU Leuven, Leuven, Belgium; c School of Chemistry and Bioscience, University of Bradford, Bradford, United Kingdom; d BCCM/ITM Mycobacterial Culture Collection, Institute of Tropical Medicine, Antwerp, Belgium; Institute for Systems Biology

**Keywords:** genomics, *Lactobacillus*, taxonomy, species delimitation, core genome

## Abstract

There are more than 200 published species within the *Lactobacillus* genus complex (LGC), the majority of which have sequenced type strain genomes available. Although genome-based species delimitation cutoffs are accepted as the gold standard by the community, these are seldom actually checked for new or already published species. In addition, the availability of genome data is revealing inconsistencies in the species-level classification of many strains. We constructed a *de novo* species taxonomy for the LGC based on 2,459 publicly available genomes, using a 94% core nucleotide identity cutoff. We reconciled these *de novo* species with published species and subspecies names by (i) identifying genomes of type strains and (ii) comparing 16S rRNA genes of the genomes with 16S rRNA genes of type strains. We found that genomes within the LGC could be divided into 239 *de novo* species that were discontinuous and exclusive. Comparison of these *de novo* species to published species led to the identification of nine sets of published species that can be merged and one species that can be split. Further, we found at least eight *de novo* species that constitute new, unpublished species. Finally, we reclassified 74 genomes on the species level and identified for the first time the species of 98 genomes. Overall, the current state of LGC species taxonomy is largely consistent with genome-based species delimitation cutoffs. There are, however, exceptions that should be resolved to evolve toward a taxonomy where species share a consistent diversity in terms of sequence divergence.

**IMPORTANCE** The *Lactobacillus* genus complex is a group of bacteria that constitutes an important source of strains with medical and food applications. The number of bacterial whole-genome sequences available for this taxon has been increasing rapidly in recent years. Despite this wealth of information, the species within this group are still largely defined by older techniques. Here, we constructed a completely new species-level taxonomy for the *Lactobacillus* genus complex based on ∼2,500 whole-genome sequences. As a result of this effort, we found that many genomes are not classified to their correct species, and we were able to correct these. In addition, we found that some published species are abnormally large, while others are too small. Finally, we discovered at least eight completely novel species that have not been published before. Our work will help the field to evolve toward a more meaningful and complete taxonomy, based on whole-genome sequences.

## Abstract (precis)

The *Lactobacillus* genus complex is a group of bacteria that constitutes an important source of strains with medical and food applications. The number of bacterial whole-genome sequences available for this taxon has been increasing rapidly in recent years. Despite this wealth of information, the species within this group are still largely defined by older techniques. Here, we constructed a completely new species-level taxonomy for the *Lactobacillus* genus complex based on ∼2,500 whole-genome sequences. As a result of this effort, we found that many genomes are not classified to their correct species, and we were able to correct these. In addition, we found that some published species are abnormally large, while others are too small. Finally, we discovered at least eight completely novel species that have not been published before. Our work will help the field to evolve toward a more meaningful and complete taxonomy, based on whole-genome sequences.

## INTRODUCTION

Since the advent of whole-genome sequencing, it has become clear that current bacterial taxonomy is not always consistent with bacterial evolutionary history. In particular, some official taxa are not monophyletic, and taxa of the same rank often differ in the diversity they represent in terms of sequence identity or shared gene content. The genus *Lactobacillus* is a prime example of both problems (1). First, it is larger than a typical bacterial family. Second, the genus is paraphyletic: the genera *Pediococcus*, *Leuconostoc*, *Weissella*, *Oenococcus*, and *Fructobacillus* are also descendants of the most recent common ancestor of *Lactobacillus*. Together, these six genera form a monophyletic taxon that is sometimes referred to as the *Lactobacillus* genus complex (LGC) (2). The LGC is an important source of bacteria with medical, food, and feed applications (1) and is a key player in the human microbiome (3, 4). Because of all this, LGC taxonomy has been the topic of much study and debate, especially during recent years (1, 5–7).

In an attempt to systematically correct inconsistencies in the official taxonomy of all *Bacteria* and *Archaea*, Parks et al. (8) recently constructed the Genome Taxonomy Database (GTDB). They built a phylogeny of a dereplicated subset of all sequenced bacterial and archaeal genomes and determined the diversity within each official taxon using a heuristic called relative evolutionary divergence (RED). With this information, they corrected taxa that were not monophyletic or that contained a diversity much above or below the average diversity of their rank. In their alternative, genome-based taxonomy, all six LGC genera were integrated into the family *Lactobacillaceae*, and the genus *Lactobacillus* was split into 15 smaller genera.

The approach of Parks et al. (8) to split/merge only taxa that are outliers in terms of diversity within their rank is appealing for the taxonomic ranks from phylum to genus, because these are relatively arbitrary and have no meaningful cutoffs in terms of diversity. The species level, however, is different. First, while there is agreement that the ranks from phylum to genus are arbitrary, there exists discussion on the possible biological meanings of the species rank. For example, one recent study found that there exists a relatively large gap between within-species and between-species genome distances; in other words, that the “genome space” shows a discontinuity corresponding to the species level (9). If this would prove to be indeed the case, it would mean that a prokaryotic species concept based on this discontinuity would hold real biological meaning. In another recent study, the hypothesis that taxa of the species rank show the property of exclusivity more than taxa of other ranks was explored (10). A taxon is exclusive if all of its members are more related to each other than to anything outside of the taxon. The authors concluded that their data falsified this hypothesis: many taxa, from many different ranks, indeed show the property of exclusivity, but that this was not more often the case for taxa of the species rank. Thus, there is currently no agreement on whether a particular biological significance should be attached to the species rank. However, a second way in which the species rank stands out is more pragmatic. In contrast to the other taxonomic ranks (e.g., the genus rank), relatively fixed similarity cutoffs to separate species are generally accepted and largely seem to correspond to the historically defined species (9, 11).

The cutoff that is commonly used to separate bacterial species based on their genome sequences, is 94 to 96% average nucleotide identity (ANI) (11, 12). Despite this cutoff being accepted as the gold standard for bacterial species delimitation, it is seldom checked for type strain genomes of validly published species and subspecies. Type strain genomes of different species are sometimes more closely related than ∼95% ANI, while type genomes of subspecies of the same species are sometimes more distantly related than ∼95% ANI. For example, it has been shown that the type strains of Lactobacillus casei and Lactobacillus zeae are too closely related to consider them separate species (13). This has led to the rejection of the L. zeae name. A related problem is that newly sequenced genomes, of type strains or otherwise, are not systematically checked for similarity against type strains. In the NCBI assembly database, uploaders of new assemblies are free to choose which species name they assign to the genomes. This has resulted in many classification inconsistencies. For example, we have previously shown that many genomes annotated as Lactobacillus casei in the NCBI database are in reality more closely related to the Lactobacillus paracasei type strain (14). Finally, it is possible that some sequenced genomes are so distantly related to all currently described species that they should be considered a new species.

In this work, we aimed to infer *de novo* species within the LGC by downloading all genomes belonging to this taxon from the NCBI assembly database and clustering them based on pairwise genome similarities. Advantages of this genome-based, *de novo* species taxonomy are as follows. (i) Future work on comparing LGC species based on genomes can be performed with a complete and nonredundant data set of exactly one representative genome per species. (ii) Comparative genomics studies of an individual species can proceed with a complete set of all genomes that are within species range of its type strain. Furthermore, we reconciled our *de novo* species with the currently established LGC species by identifying the genomes of type strains and by comparing 16S rRNA gene sequences to those of type strains. On the basis of this reconciliation, we propose changes to the official taxonomy in the form of splits or mergers of species. As a cornerstone for genome quality control, pairwise genome comparisons, and phylogeny inference, we made use of single-copy core genes (SCGs) of the LGC. The classical approach to identify SCGs in a set of genomes is to first determine the complete pangenome of the genomes, but this process is very computationally expensive, especially for data sets spanning more than a single species. Therefore, we developed a novel approach that should be easily applicable to any (large) genome data set. Briefly, we first identify candidate SCGs in a small, random subset of genomes and then extract those candidates from all genomes in the data set using HMM profiles. Our pipeline for rapid SCG extraction was implemented in progenomics, a toolkit for prokaryotic comparative genomics, and is available at https://github.com/SWittouck/progenomics.

## RESULTS

### SCGs and genome quality.

**FIG 1:**
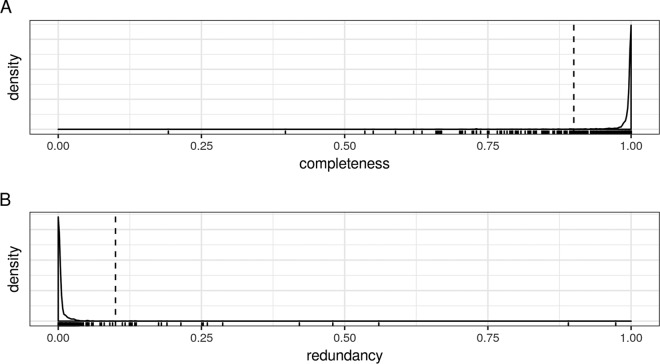
Genome quality control based on single-copy core gene (SCG) completeness and redundancy. (A) Density of genome completeness. For each genome, the percentage of SCGs that were present is shown. (B) Density of genome redundancy. For each genome, the percentage of SCGs with one or more extra copies is shown. The small vertical bars at the bottom of the graphs represent individual genomes and are shown to visualize outliers more clearly.

We downloaded all 2,558 genomes that belong to the *Lactobacillaceae* and *Leuconostocaceae* families from GenBank (see Table S1 in the supplemental material) and identified single-copy core genes (SCGs) in this data set. Our strategy for SCG extraction consisted of four steps: (i) full gene family clustering on a random subset of 30 genomes, (ii) selection of candidate SCGs from these gene families using a very soft single-copy presence threshold, (iii) search for the candidate SCGs in all genomes, and (iv) filtering of the definitive SCGs from the candidates using a fixed single-copy presence threshold. After visual inspection of the single-copy presence of the candidate SCGs in the full genome data set (see Fig. S1 in the supplemental material), we retained candidate SCGs with >95% single-copy presence. This strategy resulted in 411 SCGs *sensu lato* (“*sensu lato*” because we did not enforce 100% single-copy presence). We performed quality control of all genomes based on this set of SCGs (Fig. 1). More specifically, we determined two quality measures for each genome: completeness, defined here as the percentage of *sensu lato* SCGs that are present in the genome, and redundancy, defined as the percentage of *sensu lato* SCGs showing two or more copies in the genome. The large majority of genomes had a completeness close to one and redundancy close to zero. Enforcing a minimum completeness of 90% and maximum redundancy of 10% resulted in 2,459 high-quality genomes, or 96.1% of all genomes.

10.1128/mSystems.00264-19.1 FIG S1 Selection of definitive SCGs from candidate SCGs. Each vertical bar represents one candidate SCG (profile HMM). The *y* axis shows the percentage of all genomes a candidate SCG is present in, either in a single copy or in multiple copies. Download FIG S1, EPS file, 0.2 MB. Copyright © 2019 Wittouck et al. 2019 Wittouck et al. This content is distributed under the terms of the Creative Commons Attribution 4.0 International license.

10.1128/mSystems.00264-19.4 TABLE S1 Genomes used in this study. For each genome, the NCBI accession number, the completeness, redundancy, cluster and species name as determined by our legen pipeline, and the species name and isolation source as listed in the NCBI database are shown. Download Table S1, CSV file, 0.3 MB. Copyright © 2019 Wittouck et al. 2019 Wittouck et al. This content is distributed under the terms of the Creative Commons Attribution 4.0 International license.

Some of the genomes with low estimated completeness belonged to the genus *Sharpea*, which is part of the family *Lactobacillaceae* according to the NCBI taxonomy but seems to be phylogenetically quite distant to it (8). Other low-completeness genomes were flagged as “incomplete” in the NCBI database or were derived from metagenome data. Interestingly, some genomes belonging to Lactobacillus iners, the LGC species with the smallest genome, had very high completeness values. This suggests that genome reduction did not (significantly) influence our completeness estimates. As an extra check, we compared our completeness and redundancy estimates to the estimates available in the Genome Taxonomy Database (GTDB), where possible. There was a good correspondence between both estimates, with the exception of a small number of genomes (among which were the aforementioned *Sharpea* genomes) that had low completeness estimates by our pipeline, but high estimates in the GTDB (Table S1). This was not unexpected, since our pipeline uses LGC-specific core genes for quality estimation, while the GTDB uses a set of taxon-specific core genes.

### Pairwise genome similarities and clustering.

We compared every genome to every other genome using the core nucleotide identity (CNI), which we calculated from the supermatrix of SCGs. While average nucleotide identity (ANI) is the most commonly used genome similarity measure, it suffers from conceptual and practical drawbacks. First, since it is based on all genomic regions that are orthologous between two genomes, these regions will be different for each pairwise comparison. Thus, the type and amount of information are different across genome comparisons. Second, ANI is slow to compute for large data sets because it scales quadratically with the number of input genomes. The recently introduced fastANI (9) is orders of magnitude faster, but it suffers from the same conceptual problem and remains an approximation. Thanks to our novel approach for the rapid extraction of SCGs, computing CNI similarities becomes feasible in almost linear time.

**FIG 2:**
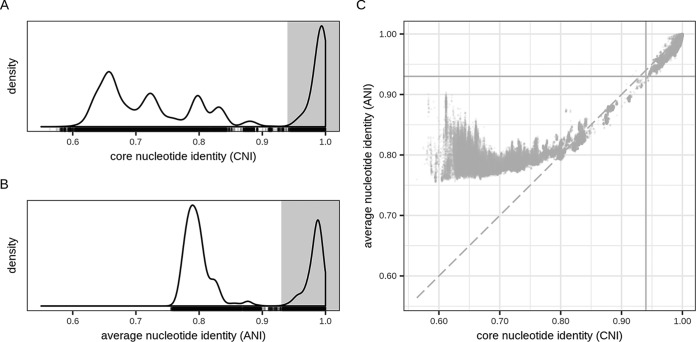
Pairwise genome distance values between LGC genomes. (A) All pairwise core nucleotide identity (CNI) similarities between LGC genomes. The gray area indicates the CNI values of >94% (the same-species range). (B) All pairwise fastANI similarities between LGC genomes. They gray area indicates the fastANI values of >93% (the hypothetical same-species range). (C) CNI versus ANI values. Only genome pairs with fastANI values of >75% are shown, since the fastANI tool does not compute values under 75%.

The density of these pairwise CNI values was strongly bimodal (Fig. 2A): there was one sharp peak with CNI values of >94%, and a flatter peak (including multiple subpeaks) with values of <90%. This indicated that two genomes were either very closely related (high CNI) or relatively distantly related (low CNI). Therefore, genomes were clustered into *de novo* species by grouping together genomes with similarities equal to or higher than 94%, using single-linkage clustering. This resulted in 239 genome clusters.

In single-linkage clustering, an object is added to a cluster when it exceeds a similarity threshold to at least one object within that cluster (as opposed to, for example, an average similarity threshold used in average linkage clustering). One problem that can arise with this strategy is that individual similarities within clusters can potentially be low, much lower than the clustering cutoff used. To assess whether this problem occurred for our clusters, for each cluster we calculated the minimum CNI between genomes within the cluster and the maximum CNI between genomes in the cluster and genomes outside of the cluster. Almost all within-cluster CNI values were higher than 94% (Fig. S2). There was only one exception: two genomes in cluster 231 had a CNI of 93.6%, but even this value was very close to the species boundary. Furthermore, all 239 genome clusters met the criterion of exclusivity: all their within-cluster CNI values were higher than their between-cluster CNI values. This included cluster 231 (later identified as Lactobacillus sakei): its highest between-cluster CNI value was 81.6% (to cluster 221, later identified as Lactobacillus curvatus).

10.1128/mSystems.00264-19.2 FIG S2 Exclusivity and transitivity of species. For each genome cluster, two numbers are shown: the minimal pairwise CNI between genomes within the cluster and the maximal pairwise CNI between a genome in the cluster and a genome from another cluster. Download FIG S2, EPS file, 0.04 MB. Copyright © 2019 Wittouck et al. 2019 Wittouck et al. This content is distributed under the terms of the Creative Commons Attribution 4.0 International license.

In addition to the CNI, we also calculated a second similarity measure between all our genomes: the fastANI similarity, which is a fast approximation of ANI. Just like the CNI distribution, the fastANI distribution was strongly bimodal (Fig. 2B). Its high-value peak covered the range 93% to 100%. A scatterplot of the CNI and fastANI similarities (Fig. 2C) showed that both measures were strongly correlated for closely related genomes (around or above the species cutoff) and that the fastANI values were generally lower than the CNIs in this range. For more distantly related genomes, with CNI of <80%, the values were clearly less correlated, and the fastANI values were (much) higher than their CNI counterparts. The CNI species delimitation cutoff of 94% roughly corresponded to a fastANI cutoff of 93%. At these cutoffs, both similarity measures were in strong agreement on whether two genomes belonged to the same species or not.

To assess whether the species level held properties absent from the other taxonomic ranks, we calculated the exclusivity of clusters at various CNI cutoff values. The results showed that for CNI clustering cutoffs between 88% and 96%, all clusters were exclusive (Fig. S3). This was not the case for most cutoffs outside of this range.

10.1128/mSystems.00264-19.3 FIG S3 Exclusivity for different CNI cutoffs. For each possible CNI cutoff for hierarchical clustering (each cutoff resulting in a different number of clusters), we computed the number of nonexclusive clusters. A cluster is exclusive if all similarities between genomes within the cluster are higher than similarities between genomes within the cluster and genomes outside of the cluster. Download FIG S3, EPS file, 0.08 MB. Copyright © 2019 Wittouck et al. 2019 Wittouck et al. This content is distributed under the terms of the Creative Commons Attribution 4.0 International license.

### Comparison with published species.

We then attempted to label all 239 genome clusters (*de novo* species) with the name of a published species. We did this in two stages: (i) identification of genomes belonging to type strains of published (sub)species (further called “type genomes”) and (ii) for the remaining clusters, extraction of 16S rRNA genes from the genomes and comparison to 16S rRNA genes from type strains of published (sub)species (further called “type 16S genes”).

In the first cluster-naming stage, we identified type genomes present in our data set (Table S2). First, we cross-referenced the strain names that were listed in the NCBI assembly database with type strain names of validly published (sub)species found on the List of Prokaryotic Names with Standing in Nomenclature (LPSN) (15, 16) or Prokaryotic Nomenclature Up-to-date (PNU) (17). This resulted in the identification of 367 type genomes within our data set, belonging to 226 unique names (species or subspecies) from 211 species. Several type genomes for the same name were often found because many type strains have been sequenced multiple times. Next, we supplemented this type genome list by manually scanning the literature for extra type genomes, including also species that were not published validly (i.e., not in an official taxonomic journal). This yielded type genomes for an additional 11 species. There were different reasons why these manually identified type genomes had not been detected by our automated approach. Some of them were not represented on LPSN and PNU because they were not validly published. For other type genomes, their species were validly published, but too recently for them to be already on LPSN or PNU. Then there were some special cases. Lactobacillus nuruki (18) was a validly published name but was not listed on LPSN or PNU, probably because its genus name was misspelled as “Lactobacilus” in the NCBI database and in the paper. Convivina intestini was missed by our automated approach because we did not include this genus in our search, while it does appear to be part of the LGC, more specifically of the family *Leuconostocaceae* (16). The Lactobacillus musae type genome was missed because its type strain name (strain 313) consists only of numbers and was consequently filtered out by our pipeline. Finally, Leuconostoc lactis had a type genome in the NCBI database that did not pass our quality control, but we could also identify a type genome of Leuconostoc argentinum, a later synonym of Leuconostoc lactis (*Leuc. lactis*). This type genome had not been identified by our automated pipeline because it filtered out rejected (sub)species names.

10.1128/mSystems.00264-19.5 TABLE S2 Type genomes identified by this study. The NCBI assembly accession number, strain name (from LPSN/PNU/StrainInfo), full name (sometimes including subspecies), and species name of genomes identified as belonging to type strains of (sub)species are shown. Download Table S2, CSV file, 0.03 MB. Copyright © 2019 Wittouck et al. 2019 Wittouck et al. This content is distributed under the terms of the Creative Commons Attribution 4.0 International license.

Of our set of 239 genome clusters, 210 contained one or more type genomes (Table S1). The large majority of these clusters (200 of them) contained type genomes from exactly one species and could thus be given the name of that species. Ten clusters contained type genomes from two or three different species, indicating that the type strains of these species were more closely related than the 94% CNI cutoff (Table 1). These clusters were cluster 135 with Lactobacillus amylotrophicus and Lactobacillus amylophilus, cluster 141 with Lactobacillus kimchii and Lactobacillus bobalius, cluster 173 with *Lactobacillus micheneri*, *Lactobacillus timberlakei*, and *Lactobacillus kosoi*, cluster 178 with Weissella jogaejeotgali and Weissella thailandensis, cluster 179 with Lactobacillus fructivorans and Lactobacillus homohiochii, cluster 218 with Pediococcus acidilactici and Pediococcus lolii, cluster 225 with Leuconostoc gelidum, Leuconostoc inhae, and Leuconostoc gasicomitatum, cluster 233 with Lactobacillus gasseri and *Lactobacillus paragasseri*, cluster 236 with Leuconostoc suionicum and Leuconostoc mesenteroides, and cluster 238 with Lactobacillus casei and Lactobacillus zeae. We assigned the name of the species that was described first to these clusters. In one situation, two subspecies of the same species had a type genome in different genome clusters: Lactobacillus aviarius subsp. *aviarius* and Lactobacillus aviarius subsp. *araffinosus*. We named the genome cluster with the latter type strain *Lactobacillus araffinosus*.

**TABLE 1:** Inconsistencies between published and *de novo* species

Cluster	Merger or split	Type strain(s) present	Minimum CNI
135	Merger	*L. amylotrophicus*, *L. amylophilus*	1
218	Merger	*P. acidilactici*, *P. lolii*	0.981
179	Merger	*L. fructivorans*, *L. homohiochii*	0.979
178	Merger	*W. jogaejeotgali*, *W. thailandensis*	0.974
173	Merger	*L. micheneri*, *L. timberlakei*, *L. kosoi*	0.964
238	Merger	*L. casei* subsp. *casei*, *L. zeae*	0.954
236	Merger	*Leuc. mesenteroides* subsp. *mesenteroides*, *Leuc. mesenteroides* subsp. *cremoris*, *Leuc. mesenteroides* subsp. *dextranicum*, *Leuc. mesenteroides* subsp*. jonggajibkimchii*, *Leuc. suionicum*	0.952
225	Merger	*Leuc. gelidum* subsp. *gelidum*, *Leuc. inhae*, *Leuc. gasicomitatum*	0.946
233	Merger	L. gasseri, *L. paragasseri*	0.946
141	Merger	*L. kimchii*, *L. bobalius*	0.944
64	Split	*L. aviarius* subsp. *araffinosus*	1
149	Split	*L. aviarius* subsp. *aviarius*	0.953

In the second cluster-naming stage, we manually named the 29 remaining genome clusters in which we had not found type genomes (Table 2). We could assign a species name to 13 genome clusters with relative certainty because two sources of information were in agreement: (i) the NCBI species labels attached to the genomes in the cluster and (ii) matches to our in-house-constructed database of 16S rRNA genes of validly published species not yet used to name a cluster via type genomes. For these genome clusters, we included a question mark in the species name to indicate that these names are not 100% certain but rather a “best guess.” Interestingly, we labeled eight clusters as “new species” because they yielded 16S rRNA gene sequences that showed no hits to our type 16S rRNA gene database, indicating that they were different from all known and validly published species. Each of these clusters consisted of exactly one genome. Finally, for the remaining nine clusters, the available information did not allow us to make a “best guess” nor to consider them novel species with certainty. We labeled those clusters “unidentified species.” An overview of all named genome clusters can be found in Table S3.

**TABLE 2:** Genome clusters without a type strain genome^*a*^

Cluster	NCBI species	No. of 16S rRNA genes	16S hits	Species^*b*^
155	*L. backii*	28	*L. backii*, *L. iwatensis*	*L. backii* (?)
185	*L. bombi*	6	*L. bombi*	*L. bombi* (?)
4	*L. coleohominis*	1	*L. coleohominis*	*L. coleohominis* (?)
175	*L. kefiri*	12	*L. kefiri*	*L. kefiri* (?)
165	*L. panisapium*	1	*L. panisapium*	*L. panisapium* (?)
7	*Leuc. carnosum*	4	*Leuc. gelidum* subsp. *aenigmaticum*, *Leuc. carnosum*, *Leuc. rapi*	*Leuc. carnosum* (?)
157	*Leuc. citreum*	32	*Leuc. citreum*, *Leuc. palmae*, *Leuc. lactis*, *Leuc. holzapfelii*, *Leuc. gelidum* subsp. *aenigmaticum*	*Leuc. citreum* (?)
176	*Leuc. pseudomesenteroides*	25	*Leuc. gelidum* subsp. *aenigmaticum*, *Leuc. pseudomesenteroides*, *Leuc. rapi*, *Leuc. holzapfelii*	*Leuc. pseudomesenteroides* (?)
129	*P. parvulus*	1	*P. parvulus*	*P. parvulus* (?)
12	*W. ceti*	25	*W. ceti*	*W. ceti* (?)
184	*W. cibaria*	93	*W. cibaria*	*W. cibaria* (?)
11	*W. hellenica*	1	*W. hellenica*, *W. bombi*	*W. hellenica* (?)
167	*W. koreensis*	10	*W. koreensis*	*W. koreensis* (?)
164	NA	1	NA	New species 1
166	NA	1	NA	New species 2
168	NA	5	NA	New species 3
169	NA	5	NA	New species 4
192	NA	5	NA	New species 5
211	NA	1	NA	New species 6
25	NA	1	NA	New species 7
3	NA	1	NA	New species 8
206	*W. bombi*	0	NA	Unidentified species 1
207	*W. hellenica*	0	NA	Unidentified species 2
1	NA	0	NA	Unidentified species 3
132	NA	10	*L. fornicalis*	Unidentified species 4
190	NA	4	*L. musae*	Unidentified species 5
196	NA	4	*L. panisapium*	Unidentified species 6
2	NA	5	*L. cerevisiae*, *L. yonginensis*	Unidentified species 7
217	NA	1	*L. caviae*	Unidentified species 8

a *Leuc*., *Leuconostoc*; NA, not available.

b A question mark in parentheses indicates that this species is a “best guess” (see “Comparison with published genomes” in Results).

10.1128/mSystems.00264-19.6 TABLE S3 Genome clusters (*de novo* species). Cluster identifier, species name, number of genomes, minimum CNI within genome cluster, maximum CNI between genome cluster and other clusters, cluster identifier of closest neighbor cluster, and exclusivity status of genome clusters. Download Table S3, CSV file, 0.02 MB. Copyright © 2019 Wittouck et al. 2019 Wittouck et al. This content is distributed under the terms of the Creative Commons Attribution 4.0 International license.

**FIG 3:**
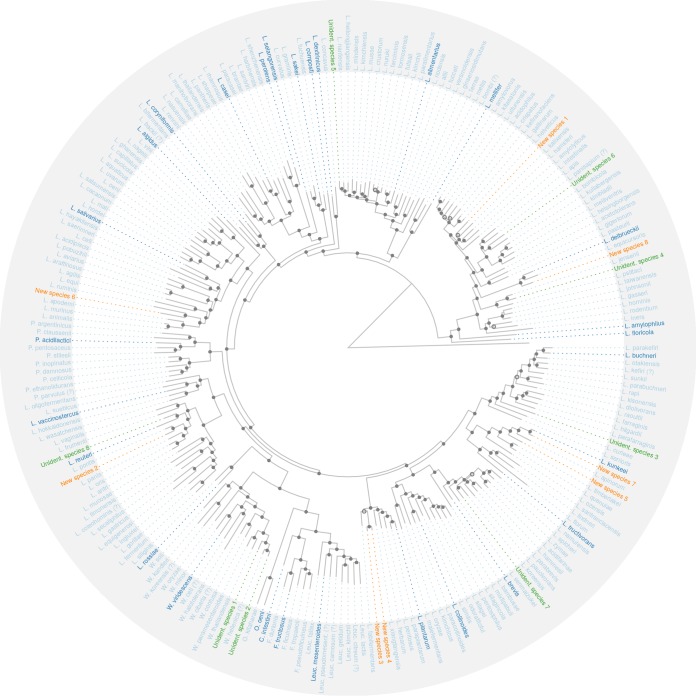
Maximum likelihood phylogenetic tree of all genome clusters of the LGC. The tree was inferred on a nucleotide supermatrix of 100 SCGs and one representative genome per species. The genes and representative genomes were selected to maximize completeness of the supermatrix. The names and terminal branches of new and unidentified species are shown in orange and green, respectively. The names of “type species” of genera or phylogroups within *Lactobacillus* (2) are shown in darker blue. Weak clades (with a bootstrap value of <70) are indicated with open circles. The root position was taken from the literature; the outgroup tip is artificial, and its branch length was chosen in order to optimally visualize the tree topology.

We inferred a maximum likelihood species tree of the LGC using one representative genome for each of the 239 genome clusters (Fig. 3). For representative genomes, we took the genomes with the largest number of SCGs, because the type strain genomes were not always of the best quality and because some genome clusters did not contain type strains. In this phylogeny, the eight new species and nine unidentified species were also included. Two of the unidentified species were situated in the *Weissella* genus; the other new and unidentified species could be found in the *Lactobacillus* genus. Some of the new/unidentified species had long “tip branches” in the tree, indicating that they were relatively far removed from the other species. We annotated the tree with the phylogroups defined by Zheng et al. (6) and predicted the lifestyle of the new species using the work of Duar et al. (2). Strikingly, the isolation source of each of the eight new species confirmed their predicted lifestyles, whether they were vertebrate-adapted, insect-adapted, or free-living bacterial species (Table 3). For nomadic phylogroups, this was trivially the case, since they can occur in any environment.

**TABLE 3:** Isolation sources, phylogroups, and predicted lifestyles of new species

Species	Isolation source (NCBI)	Phylogroup	Phylogroup lifestyle
New species 1	A *Marmota* species	*L. delbrueckii* group	Vertebrate-adapted
New species 2	Urine catheter	L. reuteri group	Vertebrate-adapted
New species 3	Kimchi	*L. plantarum* group	Nomadic
New species 4	Kimchi	*L. plantarum* group	Nomadic
New species 5	Gut of *Bombus ignitus*	*L. kunkeei* group	Insect-adapted
New species 6	Cow rumen	*L. salivarius* group	Vertebrate-adapted
New species 7	*Apis dorsata*	*L. kunkeei* group	Insect-adapted
New species 8	Human gut	*L. delbrueckii* group	Vertebrate-adapted

### Genome reclassifications.

**FIG 4:**
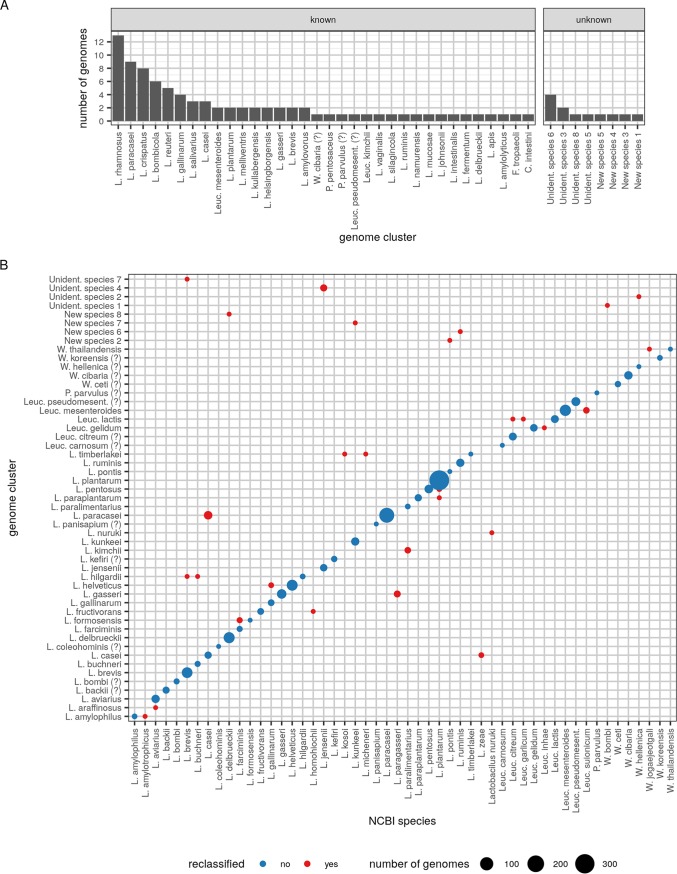
LGC genome reclassifications. (A) Classification of genomes that are currently unclassified in NCBI database, using the CNI-based genome clusters. (B) Reclassification of genomes that have an NCBI species label available but were found in a different CNI species cluster. Species names that are attached to an identical set of genomes in the NCBI and CNI classifications are not shown.

Of the 2,459 LGC genomes that passed quality control, 98 were unclassified at the species level in the NCBI database. As a direct result of our genome clustering and cluster naming pipelines, these genomes were automatically assigned to a species: either an existing one or a new or unidentified species (Fig. 4A). The most frequently identified species in this group was Lactobacillus rhamnosus, with 13 previously unclassified genomes assigned to it. Interestingly, several unclassified genomes belonged to clusters that we labeled as new or unidentified species.

Of the genomes that did have a species label in the NCBI database, 74 were reclassified to other species by our approach (Fig. 4B). Of these reclassifications, 21 were a direct consequence of the species mergers and splits we described. Next, 38 reclassifications were clear cases of genomes whose NCBI species label did not correspond to their most closely related type strain. We reclassified 20 genomes from Lactobacillus casei to Lactobacillus paracasei, 5 genomes from Lactobacillus paralimentarius to Lactobacillus kimchii, 3 genomes from Lactobacillus farciminis to Lactobacillus formosensis, 2 genomes from Lactobacillus gallinarum to Lactobacillus helveticus, 2 genomes from Lactobacillus plantarum to Lactobacillus pentosus, 1 genome from Lactobacillus brevis to Lactobacillus hilgardii, 1 genome from Lactobacillus buchneri to Lactobacillus hilgardii, 1 genome from Lactobacillus plantarum to Lactobacillus paraplantarum, and 1 genome from Leuconostoc citreum to *Leuc. lactis*. Finally, 15 genomes with a species label in the NCBI database ended up in genome clusters considered by us as new or unidentified species.

## DISCUSSION

We developed a novel approach to extract single-copy core genes (SCGs) from large genome data sets and applied it to calculate pairwise CNI as well as fastANI distances between all high-quality genomes within the LGC. We observed that for relatively closely related genomes (CNI of 80% to 100%), the fastANI similarities were lower than the CNI similarities. This could be explained by the fact that the CNI is based exclusively on conserved genes, and these genes can be expected to mutate more slowly. In contrast, fastANI is based on all homologous genome regions between two genomes and thus encompasses also recently gained, potentially fast-evolving genes. For more distantly related genomes (CNI < 80%), fastANIs were higher than CNIs. A possible explanation for this is that for these genome pairs, it becomes more difficult for an ANI calculation tool to detect the homologous regions that show lower sequence identity; the fastANI distance might thus be based only on the homologous regions with higher sequence identity because they are easier to detect. Thus, the CNI might be a better reflection of evolutionary distance because it is based on a fixed set of (prealigned) genes.

We observed a discontinuity in the density of pairwise CNI values, confirming observations made by others (e.g., reference 9). This could be taken as evidence that the species level holds real biological meaning. Alternatively, one could argue that the observed discontinuity is the result of genome sampling bias. Indeed, there have been many comparative genomics studies in recent years where the aim was to compare as many strains as possible from the same species (e.g., references 19 and 20), and this might have biased genome databases toward sets of closely related strains. However, the observation that only the CNI range of 88% to 96% shows full exclusivity of clusters is a stronger confirmation of the biological meaning of the species rank. This should be tested in more taxa to further explore whether the property of exclusivity is connected to the rank of species and to study the bacterial species question in general.

In discussing the biological significance of the species rank, we focused on species properties defined in terms of genome-genome distances. However, a number of bacterial species concepts in terms of the underlying, generative biological processes have also been proposed. In the species concept of Dykhuizen and Green (21), bacterial species are groups of strains within which recombination is common, while recombination between species is comparatively rare. Another popular species concept is the ecotype concept of Cohan and Kane (22), which states that bacterial species occupy a given ecological niche. Within a species, an innovative mutation is able to purge diversity through a vertical sweep, but this process cannot occur between different species. It is not yet clear at this point what the potential discontinuity and/or exclusivity of bacterial species would mean for the validity of the various bacterial species concepts that have been put forward.

Using a pragmatic, genome distance-based species concept, we clustered genomes of the LGC into *de novo* species using single-linkage clustering with a threshold of 94% CNI. By following this strategy, we implicitly defined a species as a set of strains for which it holds that each strain shows at least 94% core genome sequence identity to at least one other strain in the species and not to a single strain outside of the species. Using this species definition, we found that species membership in the LGC was almost transitive: all strains within a species were more than 94% similar to each other (there was only one exception to this). This has a number of interesting consequences. First, it means that, when using this species concept, species are very robust to strain/genome sampling; adding extra strains will not lead to mergers of species that were previously separated. Second, it means that for strain classification, we can calculate the similarity of the new strain to one (random) representative strain per species. When the strain is >94% similar for its core genes to one strain of a species, it will also be >94% similar to all other strains in the species. Similarly, when the strain is <94% similar to one strain of a species, it will also be <94% similar to all other strains in the species. Finally, transitivity means that clustering with single linkage or complete linkage will yield identical results. Therefore, transitivity is a very useful property to have for a given species definition and genome space. However, it should be noted that despite the fact that only one exception to transitivity was found in this work, more exceptions might be found when additional genome sequences become available in the future.

We identified situations where type strain genomes from more than one published species were found in the same *de novo* species. We argue that in these situations, the published species names should be merged. In taxonomic terms, such a merger means that the species names are considered heterotypic synonyms, i.e., names associated with different type strains but referring to the same species. Because the 94% CNI cutoff value we used (corresponding to approximately 93% fastANI) was lower than what is commonly suggested (11, 12), more species mergers than splits can be expected *a priori*. This cutoff value was chosen because it corresponded to the discontinuity we observed in the density of pairwise CNIs. A consequence of this is that for some of the mergers suggested by our pipeline, the type strain genomes will still be relatively dissimilar. Whether a merger is still preferable in these “gray area” situations depends on the species concept one wishes to use and more specifically, on the importance one attributes to the observed discontinuity in genome space for taxonomy.

Four genome clusters contained type strain genomes from different species that were very closely related (CNI > 96%). Lactobacillus micheneri and Lactobacillus timberlakei belonged to a cluster with a minimum CNI of 96.4%. These species were described in the same recent publication (23), so it is not straightforward which name should be considered the earlier synonym. Weissella thailandensis (24) and Weissella jogaejeotgali (25) were present in a cluster with a minimum CNI of 97.4%. The W. jogaejeotgali paper mentioned that its type strain was closely related to W. thailandensis, with 99.39% 16S identity. According to our genome-based comparison, *W. jogaejeotgali* should be considered a later heterotypic synonym. The type strains of Lactobacillus fructivorans (26) and Lactobacillus homohiochii (27) were present in a cluster with a minimum CNI of 97.9%. It was already known that these type strains are very closely related (28). In addition, it has been shown that other strains classified as *L. homohiochii* clearly form a separate species from *L. fructivorans* (28). Thus, in this case, the *L. homohiochii* type strain seems badly chosen. According to the International Code of Nomenclature of Prokaryotes (29), this means that *L. homohiochii* should be considered a later heterotypic synonym of *L. fructivorans* and a new type strain should be proposed for the other strains currently classified as *L. homohiochii*. Unfortunately, none of these other strains have been sequenced yet. Finally, a similar situation occurred for Pediococcus acidilactici (30) and Pediococcus lolii (31), which were in the same cluster with a minimum CNI of 98.1%. It has already been found by Wieme et al. that the P. lolii type strain DSM 19927 is an *P. acidilactici* strain (32). *P. lolii* should be considered a later synonym of P. acidilactici and if other P. lolii strains cluster separately, a new type strain and species name should be proposed for them.

Some other cases of potential species mergers were less clear-cut. Before the species Lactobacillus amylotrophicus was introduced (33), with type strain DSM 20534, its type strain had been classified to Lactobacillus amylophilus, with type strain DSM 20533 (34). We now found that these two type strain genomes were almost identical (CNI of ∼100%) at the level of their core gene sequences. However, the publication that introduced the species L. amylotrophicus (33) clearly shows that strain DSM 20534 is relatively distant from strain DSM 20533 based on a comparison of *pheS* and *rpoA* gene sequences. Thus, we suspect that a mistake was made and that both genomes are actually the same strain, while the other type strain has not yet been sequenced. It is difficult to guess which of the type strains was actually sequenced (twice), but luckily, strain DSM 20533 was also sequenced by another institute. This second DSM 20533 genome was also identical to the other two. Therefore, it is highly likely that all three genomes are DSM 20533, the *L. amylophilus* type strain, and that the *L. amylotrophicus* type strain has not been sequenced yet.

In a 2012 paper (35), it was suggested that Lactobacillus kimchii (36) and Lactobacillus bobalius (37) be considered later heterotypic synonyms of Lactobacillus paralimentarius (38). In our results, the type strains of *L. kimchii* and *L. bobalius* were in the same cluster, with a minimum CNI of 94.4%, while the *L. paralimentarius* type strain was present in a different cluster. The two clusters showed a maximum CNI value of only 93.9%. Thus, we can confirm that *L. bobalius* can be considered a later heterotypic synonym of *L. kimchii*, and we do not object to also considering both of them later heterotypic synonyms of *L. paralimentarius* since their clusters were extremely closely related. Leuconostoc gelidum (39), Leuconostoc inhae (40), and Leuconostoc gasicomitatum (41) were present in the same cluster, with a minimum CNI of 94.6%. The type strains of Leuc. gasicomitatum and *Leuc. inhae* even shared 99.5% CNI. Thus, *Leuc. inhae* should definitely be considered a later heterotypic synonym of *Leuc. gasicomitatum*. To our knowledge, this has not been suggested before. These two type strains shared 94.8% and 94.6% CNI with the *Leuc. gelidum* type strain, respectively, just on the border of being the same species.

In 2018, three Lactobacillus gasseri (42) strains were reclassified as Lactobacillus paragasseri, sp. nov. (43). We found both species in the same cluster, with a minimum CNI of 94.6%. Thus, this is a borderline case, and we do not object to *L. paragasseri* being considered a separate species. In a similar case, a subspecies of Leuconostoc mesenteroides (44) was recently introduced as the new species Leuconostoc suionicum (45). We found the type genomes of these species in the same cluster with a minimum CNI of 95.2%. This is again a borderline case.

Lactobacillus casei (46) and Lactobacillus zeae (47) were in the same cluster, with a minimum CNI of 95.4%. There has been much confusion surrounding the names L. casei, L. paracasei, and L. zeae. This is mainly the case because in 1971, strain ATCC 393 was chosen as the type strain for *L. casei* based on carbohydrate fermentation profiles (48). In 1980, this type strain was included in the Approved Lists of Bacterial Names (49). However, the strain later appeared to be relatively distant to the other historical *L. casei* strains. Subsequent proposals to change this illogical type strain (e.g., reference 50) were rejected because they violated the International Code of Nomenclature of Bacteria (13). Thus, strain ATCC 393 remained the *L. casei* type strain, which had some confusing consequences. First, the name *L. zeae* was rejected because its type strain was too closely related to strain ATCC 393. In addition, the name *L. paracasei*, whose type strain was very closely related to the historical *L. casei* strains, was kept alive. The consequence was that many of the strains that were historically classified to *L. casei* should now be reclassified to *L. paracasei*, and that all strains historically classified to *L. zeae* should now be reclassified to *L. casei* (14). In this work, we confirmed that the *L. casei* type strain and the (rejected) *L. zeae* type strain are closely related, but not so closely that it would be absurd to consider them different species.

The species *Leuconostoc garlicum* has one high-quality genome in the NCBI database, but it was reclassified to Leuconostoc lactis (51) by our pipeline. The name *Leuc. garlicum* occurs in a few publications but has never been published, validly or otherwise. Therefore, we suggest that this species name should not be validated and that these strains should classified to the species *Leuc. lactis*. Finally, we found that Lactobacillus aviarius subsp. *aviarius* and Lactobacillus aviarius subsp. *araffinosus* (52) had a CNI of 91.1%, and therefore, we suggest that the latter be renamed *Lactobacillus araffinosus* comb. nov. To our knowledge, it has never been suggested before that these should be separate species.

We labeled 13 genome clusters that contained no type genomes with a species name because their NCBI species label(s) matched their 16S rRNA hits. However, because the type strains of these species were not sequenced or their sequenced genomes were not of sufficient quality, we could not identify these *de novo* species with 100% certainty. Therefore, we propose that priority be given to (re)sequencing of these type strains. Further, we found that five species without NCBI species labels showed matches to type strains on the 16S rRNA level; these type strains should also be sequenced to be able to identify the *de novo* species with certainty. Finally, we discovered at least eight new species for which type strains should be chosen and deposited and species names should be picked.

To make it easy for people to classify their own (potential) LGC genomes against our *de novo* species taxonomy, we wrote a small classification tool. It is available at https://github.com/SWittouck/proclasp and includes a tutorial with an LGC reference database.

### Conclusions.

We constructed a genome-based species taxonomy of the *Lactobacillus* genus complex by performing single-linkage clustering on 2,459 high-quality genomes with a 94% core nucleotide identity cutoff. We found that the resulting species were discontinuous, fully exclusive, and almost fully transitive. On the basis of a comparison of the *de novo* species with published species, we proposed nine mergers of two or more species and one split of a species. Further, we discovered at least eight yet-to-be-named species that have not been published before, validly or otherwise. Finally, we have shown that our newly developed method to extract single-copy core genes allows for taxonomic studies on thousands of genomes on a desktop computer and is therefore applicable to other genera or even larger taxa. We believe that correcting the published species in the direction of our *de novo* taxonomy will lead to more meaningful species that show a consistent diversity.

## MATERIALS AND METHODS

### Downloading of genomes and gene prediction.

All genome assemblies classified to the *Lactobacillaceae* family (NCBI taxonomy id 33958) or *Leuconostocaceae* family (NCBI taxonomy id 81850) were downloaded from GenBank using the script GenBank_get_genomes_by_taxon.py that is part of the tool pyani, version 0.2.7 (53). Gene prediction was performed on all of these assemblies using Prodigal version 2.6.3 (54).

### Extraction of single-copy core genes.

To be able to rapidly extract single-copy core genes (SCGs) from our large genome data set, we devised a new approach based on seed genomes. Our strategy can be divided into four steps. In the first step, *n* seed genomes were selected completely at random, and their genes were clustered into gene families. The software program OrthoFinder (55) was used for gene family inference; it performs all-versus-all blastp (56) search, normalizes the scores for sequence length and evolutionary distances between the genomes, applies a score cutoff per gene based on its best bidirectional hits, and finally clusters the resulting weighted graph using MCL (57). This process resulted in gene families for the seed genomes only. In the second step, candidate SCGs were selected from all seed gene families by identifying gene families present in more than *k* seed genomes. The third step was the identification of these candidate SCGs in all genomes. For this purpose, the seed sequences for each candidate SCG were first aligned using MAFFT (58). Next, profile HMMs were constructed from those alignments using the hmmbuild tool of HMMER (hmmer.org). All genomes were then scanned for occurrences of the candidate SCGs with the hmmsearch tool of HMMER. Finally, a homology score cutoff was determined for each profile HMM (see the next paragraph for the details), and these cutoffs were applied to the HMMER hits to yield all “real” hits of the candidate SCGs in all genomes. In the fourth step, the final SCGs were determined by retaining only candidate SCGs with exactly one gene (copy) in at least *p* percentage of all genomes.

For the training of profile-specific hmmer score cutoffs, the following strategy was used. First, if a gene was a hit of multiple profiles (multiple candidate SCGs), only the highest scoring profile was kept. Next, profile-specific cutoffs were determined as follows. Imagine we are selecting a score cutoff for a given profile. For each genome that has at least one gene among the hits of the profile, the best-scoring gene is considered a “true” hit. All other genes (second, third, etc., best hits of genomes) are considered “false” hits. A score threshold is then determined that maximizes the F-measure of the hits; this is the harmonic mean of the precision and recall. In effect, this means that the threshold is set in such a way that as many as possible “best hits of genomes” are included in the results, while at the same time as many as possible results should be a “best hit of a genome.” The optimization was performed using the R package ROCR (59). This approach was applied separately to every profile HMM (each one representing a candidate SCG).

The SCG extraction strategy we described here was implemented in progenomics: a general, in-development toolkit for prokaryotic comparative genomics (github.com/SWittouck/progenomics). The parameter values used in this study were 30 for *n* (number of seed genomes), 25 for *k* (required number of seed genomes a candidate SCG should be present in), and 95 for *p* (required percentage of total genomes a final SCG should be present in). The software versions used were progenomics version 0.1.0, OrthoFinder version 2.1.2, blast version 2.6.0, MCL version 14-137, MAFFT version 7.407, HMMER version 3.1b2, and ROCR version 1.0.7.

### Genome quality control.

For each genome, two quality measures were calculated based on the SCGs: the completeness (percentage of SCGs present in the genome) and redundancy (percentage of SCGs having more than one copy in the genome). Only genomes with >90% completeness and <10% redundancy were retained. To compare our quality estimates to those available on the Genome Taxonomy Database (GTDB), we downloaded the metadata of the bacterial genomes of the latest GTDB release on 16 July 2019.

### Calculation of pairwise genome similarities.

For every unique pair of genomes, two similarity measures were computed: the core nucleotide identity (CNI) and average nucleotide identity (ANI). For the CNIs, multiple alignment was first performed for each SCG on the amino acid level using MAFFT version 7.407 (58) with the default parameters. Those amino acid alignments were then used as a reference to align the SCGs on the nucleotide level (reverse alignment). The aligned nucleotides were then concatenated into one large alignment (“supermatrix”). Next, pairwise nucleotide differences were calculated from this alignment using the distmat tool of EMBOSS version 6.6.0.0 (60), without correcting for multiple substitutions. Those differences were converted to similarities by subtracting them from 1. Pairwise ANIs were calculated using the tool fastANI (9), version 1.1, which quickly computes a relatively accurate approximation of ANI.

### Clustering of genomes.

Genomes were clustered into *de novo* species by joining together all genome pairs with a CNI similarity higher than 0.94. One could think of this as “nonhierarchical single-linkage clustering.” A simple algorithm to perform this clustering was implemented in R version 3.5.1 (61).

### Identification of type genomes.

To identify genomes of type strains (“type genomes”), we first downloaded assembly metadata for all of our genomes from the NCBI database using the rentrez R package version 1.2.1 (62). One of those metadata fields was the strain name of the assembled genome. Next, we compiled a list of all type strain names of validly published species of the six genera that make up the LGC. Since type strains are deposited in at least two but often many more culture collections, all of them have at least two names. To collect those type strain names, we developed an R package called tidytypes (github.com/SWittouck/tidytypes, version 0.1.0), which searches the websites LPSN (15, 16), PNU (17), and StrainInfo (63). StrainInfo was used only to find extra synonyms of type strains of species and subspecies found on LPSN and PNU. By looking up those type strain names (including their many synonyms) in the assembly metadata from NCBI (which includes a strain name field), we were able to identify type genomes in our data set on a large scale and in an automated manner. We then supplemented this list by manually identifying extra type genomes in the following way. We gathered a list of species names that were present in the NCBI assembly metadata but for which we had not already found a type genome. For each of those species, we looked up the type strain names in the original paper that described the species. We then inspected the assembly metadata of the genomes classified to that species on NCBI in an attempt to find type genomes that were missed by the automated approach (for example because the species were not validly published). The result of the automated and manual approaches was a complete list of type genomes in our genome data set.

### Assignment of species names to genome clusters.

Two different strategies were employed to assign species names to the genome clusters: one based on the type genomes present in the clusters and one based on comparisons to 16S rRNA gene sequences of type strains. First, we used the list of type genomes to assign species names to genome clusters. If the cluster contained type genomes from exactly one species, we assigned this species name to the cluster. If it contained type genomes from more than one species, we concluded that those species were closely related enough to be considered heterotypic synonyms and assigned the oldest species name to the cluster. If two clusters contained type genomes from different subspecies of the same species, we proposed upgrading the cluster with the nontype subspecies (e.g., Lactobacillus casei subsp. *tolerans*) to species status (e.g., *Lactobacillus tolerans*).

For the genome clusters that did not contain one or more type genomes, we predicted 16S rRNA genes using barrnap version 0.9 (64). For all species and subspecies names on LPSN for which we did not find a type genome in our data set, we downloaded a 16S rRNA sequence from GenBank. We then scanned the 16S sequences extracted from our genomes against this reference 16S database using the blastn tool of blast version 2.6.0 (56). We then applied a percentage identity cutoff of 98% on those hits. When a genome cluster contained one or more genomes with a match to an official species through the 16S approach, and the same species was listed in the species labels of one or more genomes, we assigned this species name to the genome cluster as a “best guess.” When a genome cluster yielded 16S genes but none of them showed a match to the reference database, we considered it a new species. Finally, some genome clusters remained that could not be identified or be considered a new species; we labeled those “unidentified.”

### Inference of species phylogeny.

For each genome cluster, the genome with the largest number of SCGs present was selected as a representative genome. We then selected the 100 SCGs that had the largest single-copy presence counts in those representative genomes. We then constructed a supermatrix with the selected genomes and SCGs, in the same way as described above in the section “Calculation of pairwise genome similarities” (including reverse alignment). Next, we trimmed columns that had gaps in more than 1% of the genomes using trimal version 1.4.rev15 (65). We inferred a maximum likelihood tree using RAxML version 8.2.11 (66) using the general time reversible model for nucleotide substitutions and the CAT method for modeling mutation rate heterogeneity across columns. We used the “-f a” option of RAxML, which combines rapid bootstrapping (67) with a slow search of the tree space starting from the bootstrap trees. The number of bootstrap trees was determined by the “N autoMRE” option, which will stop looking for bootstrap trees when they do not seem to add any extra information (68).

### Data processing and visualization.

All processing and visualization of table-format data were done in R version 3.5.1 (61) using the tidyverse set of packages, version 1.2.1 (69). Phylogenetic tree visualization and annotation were done using ggtree version 1.12.7 (70).

### Availability of data and material.

NCBI assembly accession numbers for the genomes used in this study can be found in Table S1 in the supplemental material. The code used for this study was split over four repositories. The main pipeline (called “legen,” for Lactobacillus Evolutionary Genomics) is available at https://github.com/SWittouck/legen_pipeline (version 3.1). R Markdown scripts for the data analysis, including the creation of all figures and tables, are available at https://github.com/SWittouck/legen_data_analysis (version 3.0). This repository also contains the necessary data files and can thus be run on its own. Some of the steps of the pipeline, including the extraction of single-copy core genes, were implemented in the progenomics toolkit for prokaryotic comparative genomics for maximal reusability. This toolkit is available at https://github.com/SWittouck/progenomics (version 0.1.0). Finally, searching taxonomic websites to obtain type strain names of published prokaryotic species was implemented in an R package called tidytypes, which can be found at https://github.com/SWittouck/tidytypes (version 0.1.0).
